# Unlocking the Enigma of Human Papillomavirus (HPV)-Related Multi-phenotypic Sinonasal Carcinoma: A Case With Aggressive Morphology but a Favourable Prognosis

**DOI:** 10.7759/cureus.78030

**Published:** 2025-01-26

**Authors:** Pavithra Ethirajan, Archana Lakshmanan, Ambika R. S., Rayappa C

**Affiliations:** 1 Pathology, Apollo Main Hospital, Chennai, IND; 2 Histopathology, Apollo Cancer Centre, Chennai, IND; 3 Head and Neck and Skull Base surgery, Apollo Cancer Centre, Chennai, IND; 4 Head and Neck and Skull Base Surgery, Apollo Cancer Centre, Chennai, IND

**Keywords:** adenoid cystic, hpv-related multi-phenotypic sinonasal carcinoma (hmsc), human papillomavirus (hpv), multi-phenotypic, sinonasal carcinoma

## Abstract

Human papillomavirus (HPV) is a well-known established causative agent for head and neck carcinomas, most commonly in the oropharynx. The sinonasal tract is increasingly recognised as a notable site for HPV-related carcinoma, with evidence suggesting a meaningful association with high-risk HPV. The patient is a 44-year-old female, who presented with a nasal septal growth. Initial biopsy was reported as poorly differentiated carcinoma. Subsequent excision showed malignant neoplasm with varied histomorphological patterns with areas resembling poorly differentiated carcinoma, some areas showing adenoid cystic-like features and focal pericytomatous pattern. Immunohistochemically, the neoplastic cells were diffusely positive for Pancytokeratin and p16 with a patchy expression of p63, p40, SMA, and C-KIT. The proliferation index as assessed by Ki-67 was around 35%. HPV testing by RT-PCR was positive, and the HPV-33 genotype was detected. With the given findings, a diagnosis of HPV-related multi-phenotypic sinonasal carcinoma was rendered. HPV-related multi-phenotypic sinonasal carcinoma (HMSC) shows an indolent clinical course despite an aggressive morphology. Surgery with or without radiotherapy is the recommended treatment of choice.

## Introduction

Human papillomavirus (HPV) is widely recognised as a causative factor for head and neck malignancies, particularly in the oropharynx [1]. HPV is a group of more than 200 strains, some of which are associated with a range of diseases, from benign conditions like verrucae vulgaris (common warts) and condylomata acuminata (genital warts) to malignancies, including cancers of the oropharynx, cervix, vulva, anus, and penis [2]. Cervical carcinoma ranks as the fourth most frequent malignant tumour with about 90 - 95% of cases being HPV associated [3]. Oral pharyngealand anal squamous cell carcinoma also show a 90% association with HPV infections [4,5]. Penile carcinomas are very rare malignancies with HPV association implicated in 33% of the cancers. HPV types 16 and 18 are linked to the development of these malignancies. Diagnosis can be made through the use of the p16 IHC as a surrogate marker or by performing specific HPV genotyping [6]. Neuroendocrine carcinoma of the cervix is significantly associated with HPV infection, with approximately 85.7% of cases showing the presence of the virus [7]. Similarly, oropharyngeal neuroendocrine carcinoma also exhibits a significant association with HPV [8]. However, recent findings have identified the sinonasal tract as a new critical area prone to high-risk HPV infections. This distinct type of cancer was initially identified by Bishop et al. as “HPV-related Sinonasal carcinoma with adenoid cystic features” [9,10]. This cancer type was later redefined as “HPV-related multi-phenotypic sinonasal carcinoma” (HMSC) and officially recognised in the 5th edition of the WHO classification (2022) as a distinct entity [5]. Due to the rarity of this disease, the range of its clinical and pathological characteristics remains narrowly defined. Diagnosis requires appropriate histopathological features and HPV demonstration either by PCR or in-situ hybridisation. In contrast to its worrisome and aggressive histopathological features, they follow an indolent clinical course [5]. This case study aims to contribute further to the existing literature by documenting an additional case of HMSC.

## Case presentation

**Figure 1:**
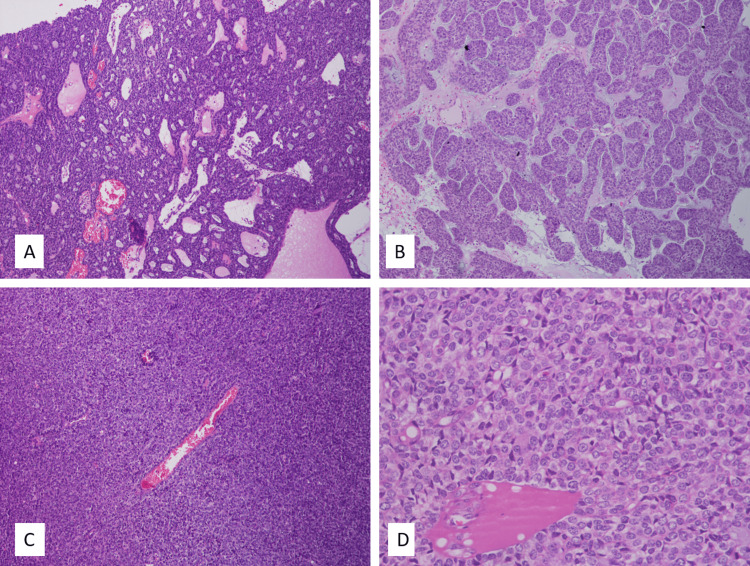
histopathological findings A) H&E (40×), B) H&E (100×) neoplasm with adenoid cystic-like and basaloid areas, C) H&E (40×), D H&E (100×) poorly differentiated areas

A 44-year-old female presented with intermittent nasal obstruction, occasional bleeding from the left nostril, and an episode of solid mass coming out from the left nostril while blowing the nose. CT PNS with contrast was done, which showed heterogeneously enhancing soft tissue density lesion in the nasal septum. The initial biopsy was reported as a poorly differentiated carcinoma. Further surgical excision revealed a malignant neoplasm characterised by diverse histomorphological patterns, including poorly differentiated regions, areas resembling adenoid cystic carcinoma, and areas displaying a pericytomatous pattern. Localised dysplasia of the surface epithelium was also observed. Patchy necrosis was seen. The overall histomorphology was suggestive of an aggressive neoplasm (Figure 1).

**Figure 2:**
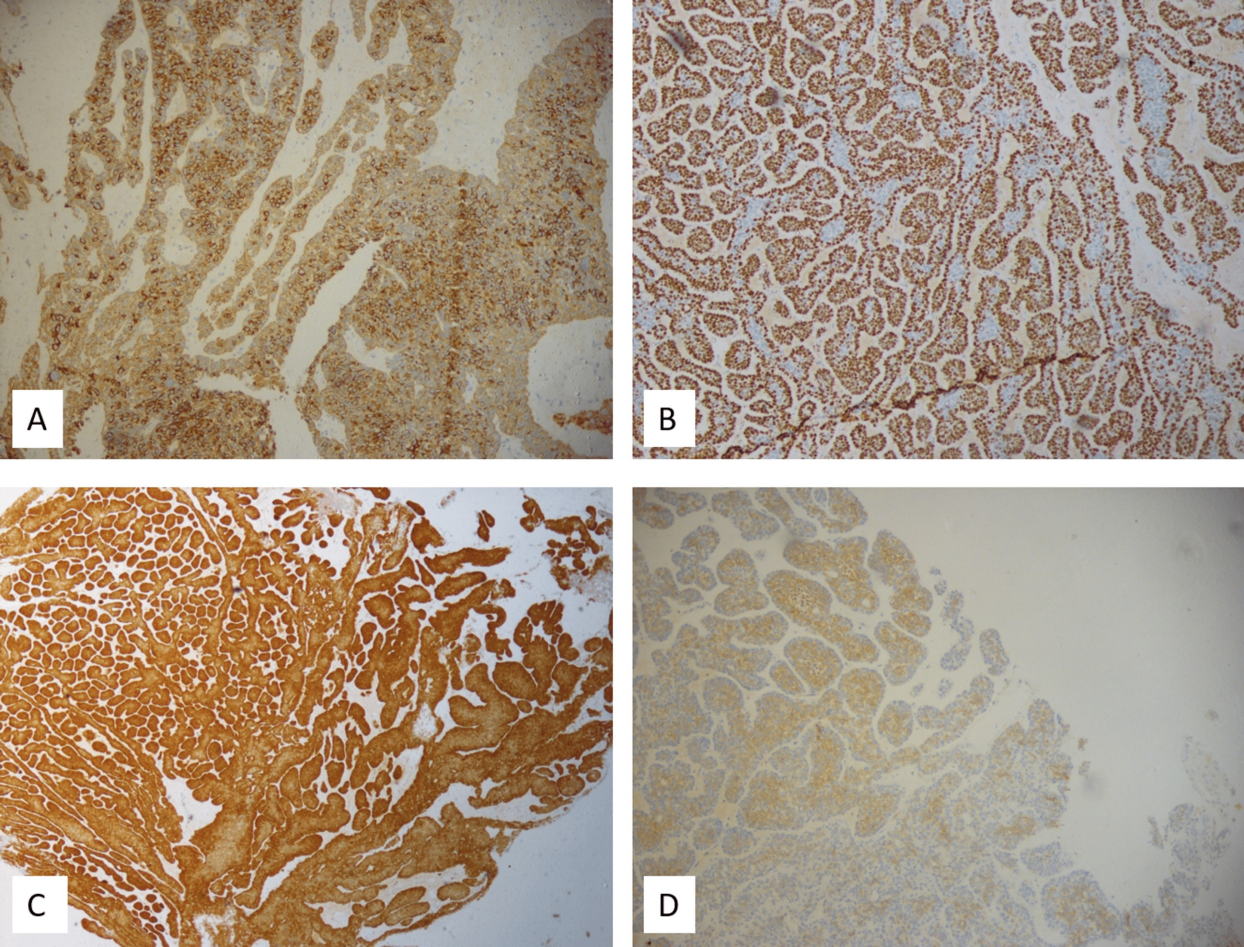
Immunohistochemical findings Neoplastic cells are diffusely positive for Pancytokeratin (A) p40, (B) P16, (C) with patchy expression of c-kit (D)

Immunohistochemical staining was done on the Ventana Benchmark XT platform. The neoplastic cells were diffusely positive for Pancytokeratin and displayed varied expressions of p63, p40, SMA, and C-KIT, which confirmed the tumour’s carcinomatous nature with both ductal and myoepithelial differentiation. The proliferation index, as indicated by Ki-67, was approximately 35%. The cells tested diffusely positive for p16, a marker indicative of high-risk HPV infection [11]. With the immunomorphological findings, a diagnosis of HMSC was favoured (Figure 2).

Real-time PCR was done at a referral laboratory, which identified HPV genotype 33, thus confirming the diagnosis of “HPV-related multi-phenotypic sinonasal carcinoma” (HMSC). The patient received radiotherapy over a period of 4 months. At the 3-year follow-up, PET CT being done every year, there were no signs of local recurrence or distant metastasis.

## Discussion

This distinctive cancer type was first identified by Bishop et al in 2012 as “HPV-related sinonasal carcinoma with adenoid cystic-like features” [12]. Following further analysis of additional cases in 2017, which included some previously misdiagnosed as adenoid cystic carcinoma, new histomorphological characteristics were observed. This led to the renaming of the condition to “HPV-related multi-phenotypic sinonasal carcinoma” to better reflect its histologic and immunophenotypic diversity. The term “multi-phenotypic” was chosen due to the tumour's morphologic heterogeneity [13]. In 2017, this cancer was listed as a provisional entity in the WHO classification, and in 2022, it was formally categorised as a definitive entity in the 5th edition of WHO [5].

This carcinoma is notably associated with high-risk HPV types, with genotype 33 being the most prevalent. Other genotypes reported include HPV 35, 16, and 56 [12,14,15]. Common symptoms include epistaxis and nasal obstruction, with some patients also experiencing nasal discharge, pain, and ocular symptoms. According to the largest case series by Bishop et al, which included 49 patients, there is a slight female predominance (57% of cases), and patients ranged in age from 28 to 90 years. The primary locations of these tumours are the nasal cavity (57%), followed by the paranasal sinuses (10%), and both regions (33%), with frequent occurrences in the maxillary and ethmoid sinuses. 36% (14 cases) of 38 patients with follow-up data experienced local recurrence and two developed distant metastases [13]. Radiologically, these tumours appear as heterogeneous enhancing lesions without distinctive features.

Histologically, they exhibit multi-phenotypic differentiation including ductal, myoepithelial, and squamous components. They are hypothesised to be originating from the terminal excretory duct at the transition to surface epithelium [12]. The presence of basaloid features and adenoid cystic-like characteristics, along with surface dysplasia and sporadic keratinisation, add complexity to the diagnosis. Differential diagnoses include the basaloid variant of squamous cell carcinoma, adenoid cystic carcinoma, NUT midline carcinoma, sinonasal undifferentiated carcinoma, and Ewing sarcoma with adenoid cystic-like features - an extended panel of immunostains, coupled with morphological assessment, aids in distinguishing these tumours.

Information on treatment and follow-up is sparse due to the rarity of this entity. Rodarte et al. have reported a case of a 65-year-old man with HMSC, positive for HPV-16 developed metastatic pulmonary nodules 23 months after surgical resection and radiotherapy. He reported this to be the case with the earliest metastasis, which supported that HPV-16 followed an aggressive course than HPV-33 [16]. Treatment typically involves surgical resection, with some patients also receiving radiotherapy [12]. There have been no reports of nodal metastasis or mortality directly linked to this disease [12].

## Conclusions

HMSC typically presents at an advanced tumour stage and exhibits aggressive morphological features. However, it paradoxically tends to follow an indolent clinical course. This discrepancy underscores the importance of accurate diagnosis, distinguishing it from other aggressive malignancies like squamous cell carcinoma, adenoid cystic carcinoma, NUT midline carcinoma, and sinonasal undifferentiated carcinoma, among others. Given that HMSC is a recently defined entity with relatively few cases documented thus far, conducting larger studies would be invaluable. These studies would enhance our understanding of the disease and facilitate the development of more effective management strategies.
